# Lysyl Hydroxylase 3 Localizes to Epidermal Basement Membrane and Is Reduced in Patients with Recessive Dystrophic Epidermolysis Bullosa

**DOI:** 10.1371/journal.pone.0137639

**Published:** 2015-09-18

**Authors:** Stephen A. Watt, Jasbani H. S. Dayal, Sheila Wright, Megan Riddle, Celine Pourreyron, James R. McMillan, Roy M. Kimble, Marco Prisco, Ulrike Gartner, Emma Warbrick, W. H. Irwin McLean, Irene M. Leigh, John A. McGrath, Julio C. Salas-Alanis, Jakub Tolar, Andrew P. South

**Affiliations:** 1 Division of Cancer Research, University of Dundee, Dundee, United Kingdom; 2 Stem Cell Institute and Pediatric Blood and Marrow Transplantation, University of Minnesota, Minneapolis, Minnesota, United States of America; 3 The Centre for Children’s Burns Research, Queensland Children’s Medical Research Institute, Royal Children’s Hospital, The University of Queensland, Brisbane, Australia; 4 Department of Dermatology & Cutaneous Biology, Thomas Jefferson University, Philadelphia, Pennsylvania, United States of America; 5 Centre for Dermatology and Genetic Medicine, Division of Molecular Medicine, Colleges of Life Sciences and Medicine, Dentistry & Nursing, University of Dundee, Dundee, United Kingdom; 6 St. John's Institute of Dermatology, King's College London (Guy's Campus), London, United Kingdom; 7 Basic Sciences Department, Medicine School, University of Monterrey, Monterrey, Mexico; University of Vienna, Max F. Perutz Laboratories, AUSTRIA

## Abstract

Recessive dystrophic epidermolysis bullosa (RDEB) is caused by mutations in *COL7A1* resulting in reduced or absent type VII collagen, aberrant anchoring fibril formation and subsequent dermal-epidermal fragility. Here, we identify a significant decrease in PLOD3 expression and its encoded protein, the collagen modifying enzyme lysyl hydroxylase 3 (LH3), in RDEB. We show abundant LH3 localising to the basement membrane in normal skin which is severely depleted in RDEB patient skin. We demonstrate expression is in-part regulated by endogenous type VII collagen and that, in agreement with previous studies, even small reductions in LH3 expression lead to significantly less secreted LH3 protein. Exogenous type VII collagen did not alter LH3 expression in cultured RDEB keratinocytes and we show that RDEB patients receiving bone marrow transplantation who demonstrate significant increase in type VII collagen do not show increased levels of LH3 at the basement membrane. Our data report a direct link between LH3 and endogenous type VII collagen expression concluding that reduction of LH3 at the basement membrane in patients with RDEB will likely have significant implications for disease progression and therapeutic intervention.

## Introduction

Epidermolysis bullosa (EB) is a heterogeneous group of inherited disorders that can be either localized within skin or systemic in presentation and severe cases are often life-threatening [1]. Technological and innovative approaches to therapies such as allogeneic cellular therapy [2,3], gene therapy [4], and protein therapy [5] all offer substantial promise but current limitations in both our understanding of each individual disease and the early stage of clinical development suggest that several approaches are needed to be combined to realize a cure [6]. One particular sub-type of EB, RDEB, has garnered the lion’s share of therapeutic development due to the devastating phenotype [7]. A wide variety of loss-of-function mutations in the gene encoding type VII collagen, *COL7A1*, cause RDEB [8] and lead to a spectrum of severity encompassing skin blistering with mucosal involvement, extensive scarring in response to wound healing, nail dystrophy, alopecia, mitten deformities of the hands and feet, and a significant risk of developing life threatening skin cancer [9].

Bone marrow transplantation is currently the only systemic novel therapy for treating RDEB and recent trials demonstrate clinical benefit for the majority of patients who successfully tolerate immunomyeloablative chemotherapy and allogeneic stem-cell transplantation [2]. However, this study demonstrated that although increased type VII collagen is evident in patient skin after transplantation, anchoring fibrils (the major structural component resulting from type VII collagen processing) are absent some 100 days post procedure [2] suggesting that the mere presence of increased type VII collagen was insufficient to recapitulate the ultrastructural features of fully functional skin in RDEB patients receiving therapy. This indicates that further refinements to the procedure or additional interventions are required for a complete cure.

Integrity of epithelia relies on multi-protein adhesion complexes that secure intracellular components to underlying tissue. In skin this is achieved by interactions between the basal keratinocyte cytoskeleton, hemidesmosome complexes at the dermal-epidermal junction and the underlying anchoring fibrils. Anchoring fibrils are supramolecular structures primarily composed of type VII collagen [10] that originate and terminate within the basement membrane [11] tethering this superstructure to collagen bundles within the dermis [12]. Synthesis and processing of mature collagen molecules involves many post-translational modifications that ensure correct intramolecular association between three polypeptide chains as well as intermolecular interaction between collagens of the same and different species dependent on tissue and functional context (for review see [13]). Type VII collagen is first organized as a homotrimer [14] within the cell which is subsequently secreted and cleaved at the C-terminal domain to yield antiparallel dimers which are in turn bundled together to form anchoring fibrils [15]. Other post-translational modification and processing events that lead to mature anchoring fibril formation have yet to be reported but as type VII collagen retains structural attributes present in all collagens it can be assumed common enzymatic modification pathways exist [16]. The central collagenous domain follows a classical Gly-X-Y repeat sequence where amino acids at position Y are frequently modified through a variety of enzymatic processes necessary for proper function. Hydroxylation and subsequent glycosylation of lysine residues at position Y is emerging as an essential post-translational modification (reviewed in [17]) as evidenced by lethality of knockout mice [18–20] and human diseases associated with mutation in the enzymes which catalyze this reaction, lysyl hydroxylase (LH) [21–23]. Different LH isoforms exist; LH1, LH2a, LH2b and LH3, but only LH3 has the ability to glycosylate hydroxylysine residues and is the only LH to be secreted and present in the extracellular space [24–26]. Reduction of LH3 causes deleterious changes to the deposition and organization of extracellular matrix in skin and from skin derived fibroblasts [27]. Here, we identify a significant reduction of LH3 in RDEB skin and investigate the relationship between LH3 and type VII collagen.

## Results

### Lysyl hydroxylase 3 localizes to epidermal basement membrane and is reduced in patients with recessive dystrophic epidermolysis bullosa

**Fig 1:**
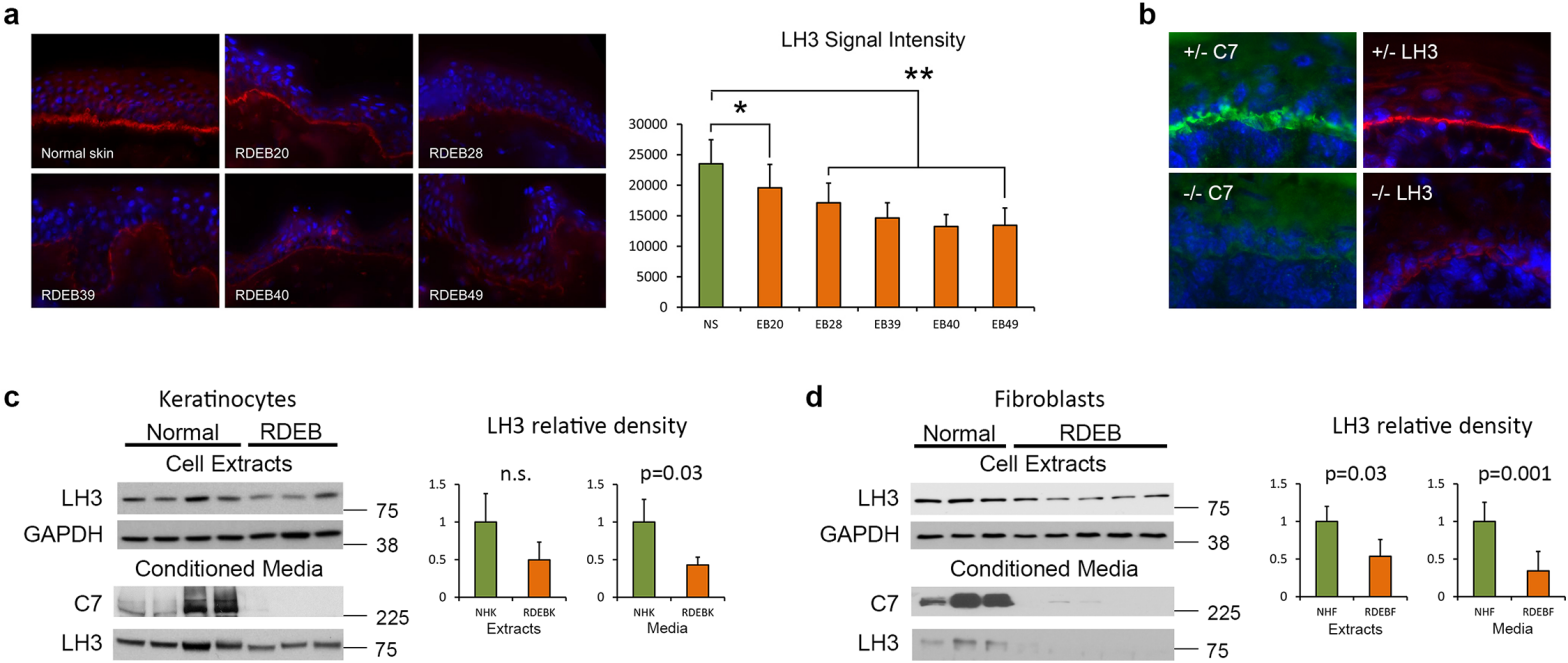
LH3 localizes to the basement membrane and is reduced in RDEB. **(a)** LH3 immunohistochemistry reveals bright linear reactivity at the basement membrane in normal skin (top left panel) which is discontinuous and greatly reduced in RDEB skin. Graph shows morphometric quantification of LH3 signal intensity, +/- standard deviation. * = p < 0.01, ** = p < 0.0005, students-T test. (**b**) Type VII collagen immunohistochemistry shows bright linear reactivity at the basement membrane in *COL7A1* +/- newborn murine skin (+/- C7, top left panel) which is absent in *COL7A1* -/- newborn murine skin (bottom left panel). LH3 immunohistochemistry shows bright linear reactivity at the basement membrane in *COL7A1* +/- murine skin (top right panel) which is reduced in *COL7A1* -/- murine skin (bottom right panel). (**c**) LH3 protein is reduced in cell extracts and significantly reduced in conditioned media from RDEB keratinocytes (RDEB1K, RDEB14K and RDEB2K) compared with normal keratinocytes (NHK, K16 and K17). (**d**) Fibroblast cultures isolated from RDEB patients express significantly less LH3 compared with cultures of normal fibroblasts (NHF). Graphs show average densitometry of LH3 protein normalized to GAPDH (using ImageJ version 1.48, imagej.nih.gov/ij/) for RDEB or normal controls. p values calculated using student-T test and error bars show +/- standard deviation.

To investigate a link between the altered dermal architecture in RDEB [28,29] and a reduction of the essential collagen modifying enzyme lysyl hydroxylase 3 (LH3), we followed up our previous gene expression profiling of cultured keratinocytes [30] that shows *PLOD3* (encoding LH3) is significantly reduced in RDEB (S1 Table). Antibodies raised against LH3 revealed striking localization to the dermal-epidermal junction in normal skin which was significantly reduced in RDEB patients (Fig 1A). Similarly, a reduction in LH3 localization at the basement membrane is also evident in *COL7A1* -/- epidermis isolated from a humanized murine model of RDEB (Fig 1B; for details on creation of this novel RDEB model see S1–S3 Figs). The reduction in expression of *PLOD3* and LH3 was confirmed in human keratinocytes derived from RDEB patients by Q-PCR (panel a in S4 Fig) and western blotting of both total cell lysate and secreted proteins using antibodies raised against LH3 (Fig 1C). Although gene expression analysis of cultured fibroblasts did not reveal changes between RDEB and non-RDEB fibroblasts at the level of mRNA ([28]; panel b in S4 Fig), western blotting identified a depletion of secreted LH3 from RDEB fibroblasts (Fig 1D). Overall these data show a significant reduction in LH3 secretion in RDEB *in vivo* and *in vitro*.

### Lysyl hydroxylase 3 interacts with type VII collagen *in vivo* and co-localizes with the central collagenous domain of type VII collagen by postembedding immunoelectron microscopy

**Fig 2:**
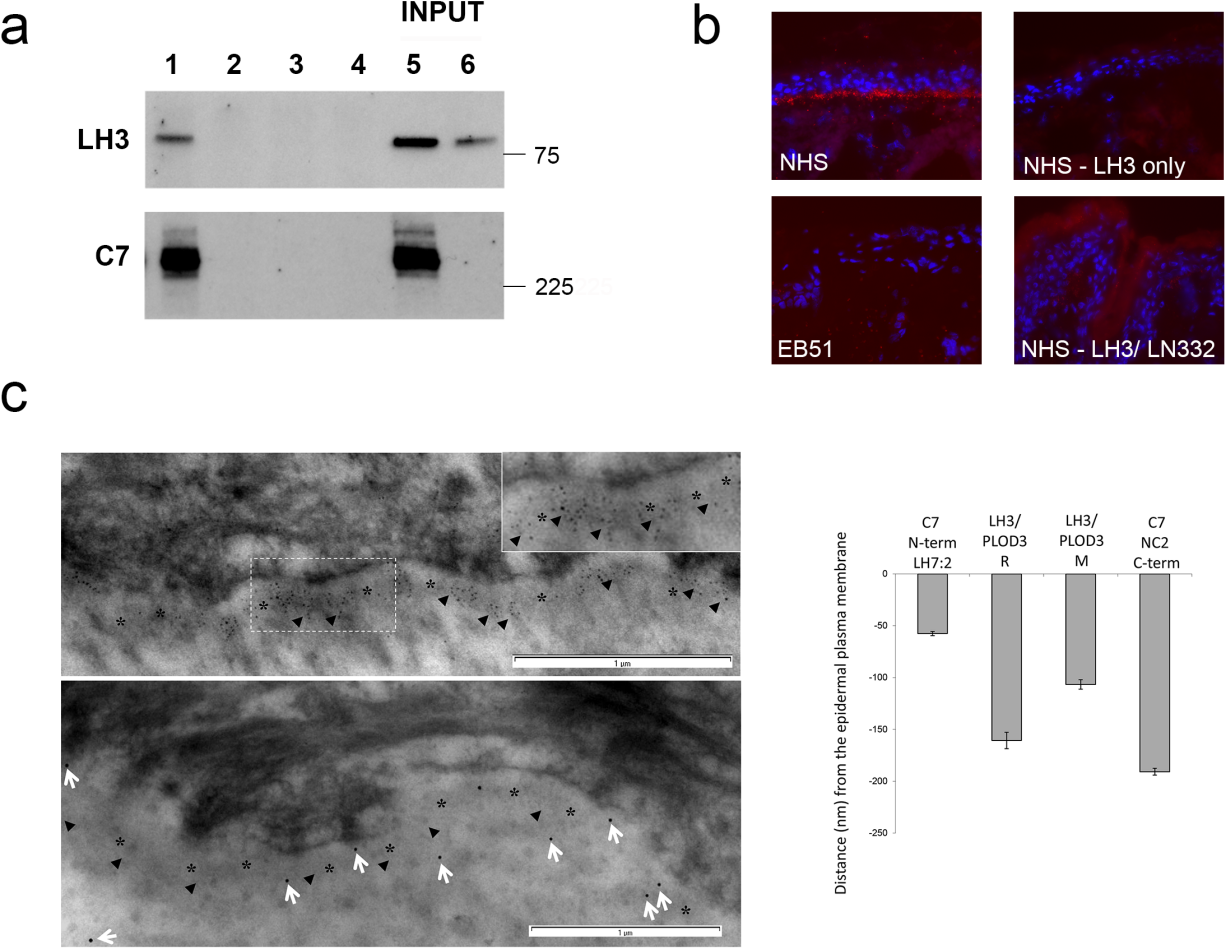
Type VII collagen binds LH3 *in vitro* and *in vivo* at the basement membrane in normal skin. **(a**) LH3 interacts with type VII collagen (C7) *in vitro*. Cell extracts from RDEB keratinocytes retrovirally expressing COL7A1 were immunoprecipitated (IP) with a C7 antibody (lane 1) and immunoblotted for LH3 co-precipitation. Controls include a non-specific IgG (lane 2), protein G beads alone (no antibody, lane 3) or C7 IP in COL7A1 null keratinocyte extracts (lane 4). Lanes 5 and 6 represent the inputs from COL7A1 expressing and null keratinocyte extracts respectively. (**b**) Proximity Ligation Assay demonstrates the close interaction of antibodies raised against LH3 and type VII collagen *in vivo* at the basement membrane in normal skin (top left panel, red signal) which is greatly reduced or absent in RDEB skin, with LH3 antibody only or with antibodies against LH3 and LN332 γ-2 chain. (**c**) Postembedding immunoelectron microscopy of human skin shows immunogold labeling beneath epidermal tissue using the LH7.2 mouse monoclonal antibody that recognizes the type VII collagen C-terminus (C7, upper panel, 5nm particles, arrowheads). LH3 polyclonal antisera (LH3, lower panel, 10nm particles, white arrows) localized beneath the epidermal tissue in an overlapping distribution close to anchoring fibrils (arrowheads, lower panel). Graph shows morphometric analysis of distance from plasma membrane (indicated by *) of immunogold particles. There were statistically significant differences (p < 0.01) observed between the depth of the two PLOD3 epitopes and both collagen VII epitopes. LH3/PLOD3 R = rabbit polyclonal antibody, M = mouse monoclonal antibody.

In agreement with a recent report using stable isotope labeling by amino acids in cell culture (SILAC) analyses of affinity-purified type VII collagen complexes [31] we show an interaction between LH3 and type VII collagen *in vitro* by immunoprecipitation and western blotting (Fig 2A). Type VII collagen immunoprecipitates from RDEB keratinocytes retrovirally transduced with *COL7A1* showed clear reactivity for LH3 when immunoblotted with an LH3 specific antibody (Fig 2A). *In situ* proximity ligation assay (PLA) confirmed a direct interaction between LH3 and type VII collagen at the basement membrane *in vivo* in normal skin which was reduced or absent in RDEB skin (Fig 2B). PLA with antibodies against LH3 and another basement membrane component, the γ2-chain of laminin 332, did not show similar reactivity. Co-localization was apparent using dual immunofluorescence (IF) with antibodies raised against type VII collagen and LH3, and the data also suggests a direct relationship between the amount of type VII collagen immunoreactivity and LH immunoreactivity at the basement membrane (not shown). Postembedding immunoelectron microscopy and localization morphometric measurement of antibodies raised against the N and C termini of type VII collagen as well as the C-terminus of LH3 demonstrated immunogold labeling beneath epidermal tissue where LH3 was localized to two main places; over the lamina densa/basement membrane beneath hemidesmosomes and in the sub-lamina densa region in an overlapping distribution close to anchoring fibrils (Fig 2C; for antibody details see Table 1). There were statistically significant differences (P<0.01) observed between the depth of the two LH3 epitopes and both type VII collagen epitopes suggesting that LH3 co-localizes with the central collagenous domain of type VII collagen *in vivo* (Fig 2C).

**Table 1:** Antibodies used in immunoelectron microscopy.

Clone or antibody name	Antigen or epitope recognized	Species and Immunoglobulin subtype	Dilution	Number of gold particles examined	Reference
Collagen VII LH7.2	CollagenVII N-terminal	Mouse IgG	1 in 10	294	[43]
CollagenVII NC-2domain	CollagenVII C-terminal	Rabbit polyclonal	1 in 200	253	[11]
LH3/PLOD3 (M) Mouse monoclonal 60058-1-Ig	LH3/PLOD3 antibody	Mouse IgG2a	1 in 10	207	Proteintech Group, Chicago, IL
LH3/PLOD3 (R) Rabbit polyclonal 11027-1-AP	LH3/PLOD3 antibody N-terminal	Rabbit polyclonal	1 in 20	250	Proteintech Group, Chicago, IL
Rabbit polyclonal pre-immune serum	Pre immune	Rabbit	1 in 200	No BMZ significant / specific labeling	[11]
NS0 myeloma cell line supernatant	None	Mouse IgG	neat	No BMZ significant / specific labeling	AbD Serotec, Raleigh, NC
Anti-human HLA MHC class I MCA485	Anti-human HLA MHC class I antibody	Mouse IgG	1 in 20	No BMZ significant / specific labeling	AbD Serotec, Raleigh, NC

### Endogenous type VII collagen regulates LH3 expression in RDEB keratinocytes

**Fig 3:**
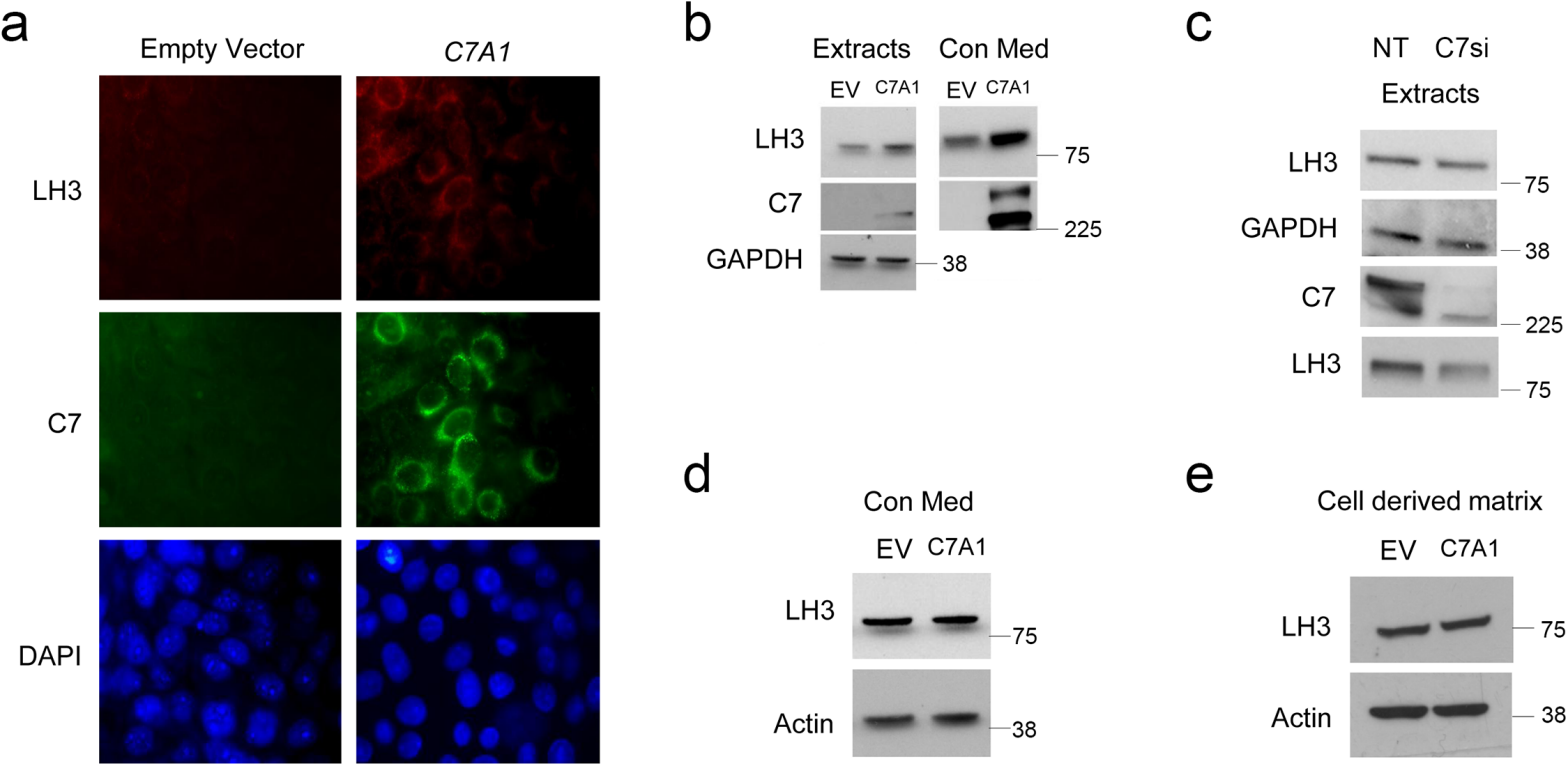
Endogenous but not exogenous type VII collagen increases LH3 expression in RDEB keratinocytes. **(a**) Retroviral delivery of type VII collagen in RDEB14 keratinocytes increases LH3 expression by immunofluorescence. (**b**) Type VII collagen increases LH3 in RDEB14 keratinocytes in cell extracts and conditioned media by immunoblotting. (**c**) siRNA depletion of *COL7A1* reduces LH3 expression in normal keratinocytes compared with non-targeting control (NT). (**d**) RDEB14 keratinocytes grown in conditioned media collected from RDEB14 keratinocytes overexpressing type VII collagen do not have increased LH3 expression compared with conditioned media collected from RDEB14 keratinocyte empty vector controls. (**e**) RDEB14 keratinocytes grown on cell matrix derived from type VII collagen overexpressing RDEB14 keratinocytes do not show increased LH3 expression compared with empty vector control treated cells. Empty Vector or EV = empty vector cells, C7A1 = *COL7A1* over expressing cells.

Next, we analyzed the association between C7 expression and LH3 by looking at whether type VII collagen could influence levels of LH3 expression. Using viral mediated over-expression we show that full length type VII collagen increases LH3 expression in RDEB keratinocytes (Fig 3A and 3B). siRNA knockdown of either endogenous (Fig 3C) or virally expressed *COL7A1* (not shown) concomitantly led to reduced LH3 expression and secretion. A reciprocal relationship was not evident as *PLOD3* siRNA knockdown did not affect type VII collagen levels (S5 Fig). To determine whether LH3 expression correlates with endogenous or exogenous type VII collagen in RDEB keratinocytes we cultured cells in the presence of matrix or conditioned media derived from type VII collagen over-expressing cells and saw no change in LH3 levels (Fig 3D and 3E).

### Bone marrow transplantation increases type VII collagen but does not change LH3 at the basement membrane in RDEB patients

**Fig 4:**
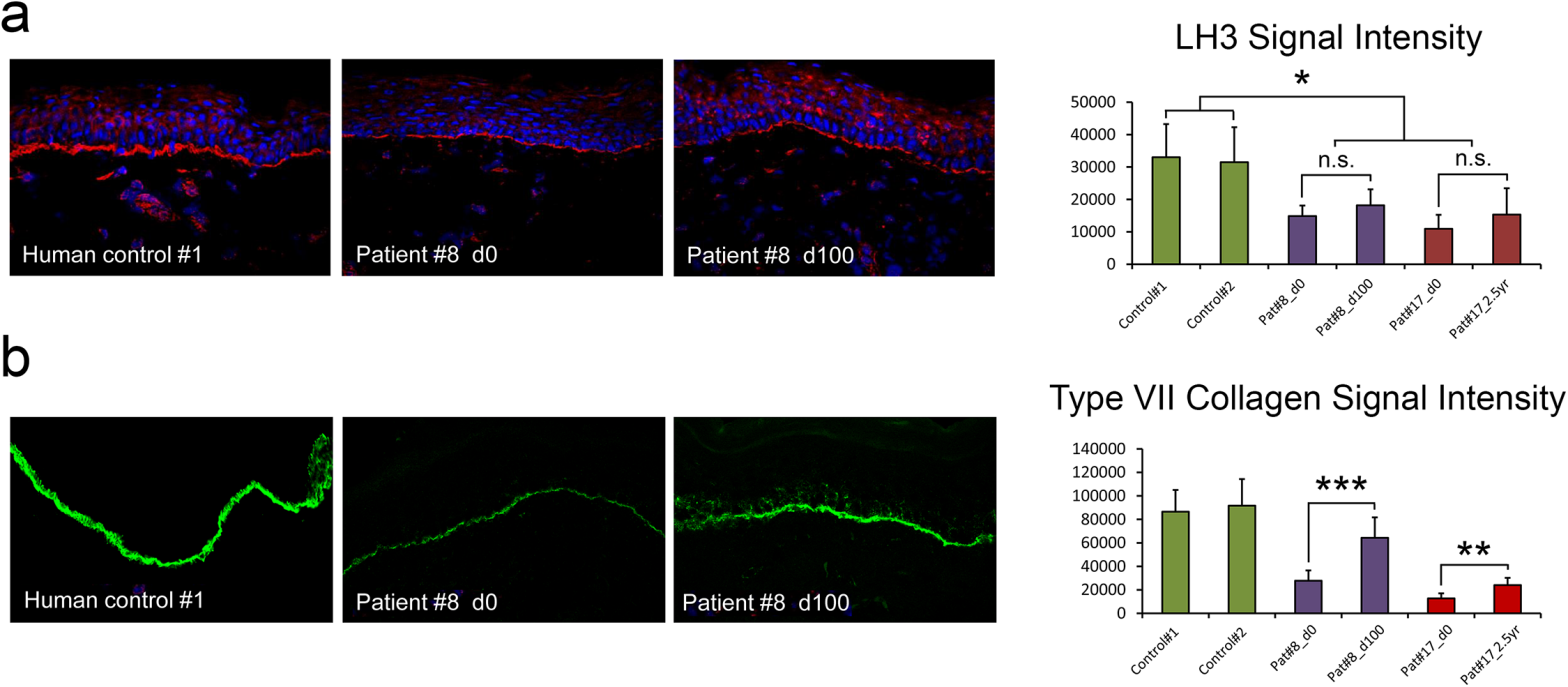
Bone marrow transplantation increases type VII collagen but does not change LH3 at the basement membrane in RDEB patients. **(a**) LH3 immunoreactivity (red) does not change after bone marrow transplantation in two RDEB patients. (**b**) Type VII collagen expression (green) is increased after bone marrow transplantation. d = day, yr = year. Graph shows morphometric quantification of LH3 and type VII collagen signal intensities, +/- standard deviation. * = p < 0.001, ** = p < 0.0001, *** = p < 0.00001, students-T test.

The above data demonstrates that endogenous type VII collagen regulates LH3 expression and suggests that therapies designed to deliver type VII collagen to RDEB patients will not alter LH3 levels unless a significant proportion of cells within the patient endogenously express type VII collagen. Therefore, in order to determine whether this is the case we assessed expression of LH3 in RDEB patients after bone marrow transplantation where it has been demonstrated in animal studies that a small number (0.5%) of donor bone marrow-derived cells home to skin wounds and secrete type VII collagen [32]. We used IF and morphometric quantification to determine the extent of immunoreactivity to LH3 specific and type VII collagen specific antibodies at the basement membrane of patient skin. Although type VII collagen was significantly increased as a result of transplantation, LH3 levels remained constant (Fig 4) indicating that indeed, in patients with exogenous type VII collagen delivery, LH3 levels did not increase.

## Discussion

Our results reveal a collagen modifying enzyme which is essential for proper basement membrane formation [19] and extracellular matrix organization [27], is present at the basement membrane in skin and is significantly reduced in patients with RDEB (Figs 1 and 4). The data are consistent with a reduction in secreted LH3 in response to reduced or absent endogenous type VII collagen (Figs 1–4). Given the reported importance of LH3 in deposition and organization of extracellular matrix [27] and that LH3 modifies proteins in the extracellular space [24], reduction of LH3 likely contributes to the altered dermal microenvironment composition observed in RDEB and in response to type VII collagen depletion in normal fibroblasts [28,29,33]. In this respect it is important to note that two studies by Kuttner and colleagues using a proteomics approach identified that LH3 was increased in RDEB fibroblasts [29,31]. We attribute the observed differences in LH3 expression to a combination of different analytical approaches, initial culture densities and timing of isolation. We also note that although cultured cells are an excellent model of human skin they themselves have limitations even though the significant reduction of LH3 in RDEB skin *in vivo* is supported by our *in vitro* studies. Indeed, the recent proteomic approach [31] identified a reduction in type IV collagen which was not altered in our cohort of RDEB patients *in vitro* or *in vivo* (not shown). This observation is well established as type IV collagen is reported to be unchanged in RDEB skin by numerous, independent studies utilizing type IV collagen antibodies as a diagnostic tool [34–36]. Furthermore, the lack of difference in type IV collagen expression in RDEB rules out a link between this basement membrane associated collagen and LH3 in RDEB, which might be implied by the observation that mice lacking LH3 fail to develop correct basement membrane as a result of impaired type IV collagen secretion [18]. Here, we show that reduction of LH3 in RDEB is a result of reduction or absence of type VII collagen, rather than alterations in type IV collagen.

One deficiency of our study is that we do not demonstrate a direct functional link between reduced LH3 levels in RDEB and the impact this may have on the altered dermal microenvironment observed in patient skin. We are pursuing this aspect as part of a larger study dissecting the role of extracellular LH3 in skin, work which is beyond the scope of the current investigation. However, given the direct relationship between LH3 levels and glycosylation activity in murine organs [24] we expect a similar relationship to exist in human skin and that a reduced level of activity in RDEB will have similar consequences to those described in experimental settings. Consistent with multiple mechanisms regulating LH3 expression and secretion, two secretory pathways have been reported; a higher molecular weight N-glycosylated form of LH3 is secreted via the ER/Golgi pathway into the extracellular space and serum, while LH3 found at the cell surface appears to bypass processing in the Golgi [26]. We have confirmed that both keratinocytes and fibroblasts isolated from skin secrete the higher molecular weight N-glycosylated form (not shown). Our observations of reduced LH3 in RDEB is intriguing, especially in light of our findings that LH3 interacts with type VII collagen at the epidermal basement membrane (Fig 2). Glycosyltransferase activity and cellular secretion is specific to LH3 and the latter is reported to be dependent on the former [26]. Given the importance of glycosylated hydroxylysines in collagen IV tetramerization [37]) and the demonstration that LH3 glucosylation activity within the collagenous domain of adiponectin is essential for its oligomerization [38], it would follow that LH3 has a crucial role in the formation of anchoring fibrils through its modification of type VII collagen, a potential substrate of LH3. Certainly, this would fit with the observation that LH3 glycosyltransferase activity is essential for basement membrane formation [19] and the absence of mature anchoring fibrils in patients receiving bone marrow transplantation [2]. Again, these aspects are being pursued as a separate, larger study.

A number of disease modifiers in RDEB have been proposed [39–41] and although no single pathway has been replicated in multiple patient cohorts [42] it is clear there is significant phenotypic diversity between members of the same family with identical *COL7A1* mutations. It is therefore tempting to speculate that polymorphisms in the *PLOD3* gene which result in reduced expression or activity may well be a disease modifier. Further work will be needed to confirm this but it is interesting to note that although LH3 levels were consistently low in RDEB cells in culture there was considerable variation between patients (Fig 1).

Finally, our observations that LH3 levels do not change after bone marrow transplantation even when an increase in type VII collagen is evident (Fig 4) raises important questions. Firstly, although murine data demonstrate that full length type VII collagen can be secreted and correctly processed by donor marrow cells after transplantation, it has not been proven that the increase in type VII collagen in human RDEB patient skin after transplantation is a result of donor marrow cells or represents an increase in patients own type VII collagen, as has been suggested for other trials involving allogenic cell transfer [3]. Such a scenario might suggest that full length, correctly processed type VII collagen is required for increased LH3 expression, whereas if the increased type VII collagen derives from a small number of donor marrow cells this might suggest that although type VII collagen is able to home to the basement membrane zone, as has been demonstrated after injection of recombinant type VII collagen protein [5], the overall number of donor cells does not impact the expression and secretion of LH3 from the patients own cells. Further work is required to determine whether *PLOD3* expression and/or LH3 secretion can be enhanced pharmacologically and whether this will have any additional clinical benefit to RDEB patients receiving type VII collagen-based therapies.

## Materials and Methods

### Skin tissue

Samples were obtained after informed, written consent, and were from unremarkable skin from a non-specialized site obtained after routine surgical or diagnostic procedures. This study has been approved by the appropriate research ethics committees (University of Minnesota IRB 0911M74035; UK MREC 05/Q0603/9; The Royal Children’s Hospital and Health Services District Human Research Ethics Committee (2008/111), University of Queensland) and in accordance with the Helsinki declaration.

### Antibodies

The antibodies used in this study were: mouse monoclonal LH7:2 used at 1:2 dilution as described for IEM [43] and 1:800 for proximity ligation assay (PLA); Rabbit polyclonal antisera to type VII collagen raised against the NC1 domain (Moravian Biotechnology, Brno, Czech Republic); mouse monoclonal antibody against type VII collagen #611607 (lot 01591 30625) 1:200 dilution used for IF (BD Biosciences, San Jose, CA); PLOD3 (11027-1-AP, Lot 00001261) rabbit polyclonal used at 1:5 dilution for IEM and 1:10–20 for IMF (ProteinTech Group, Chicago Ill, USA); PLOD3 (60058-1-Ig) mouse monoclonal used at 1:500 dilution for Western blot (ProteinTech Group); Laminin-332 (γ2 chain) mouse monoclonal (MAB19562, Millipore, Billerica, MA); GAPDH mouse monoclonal (G8795, Sigma Aldrich, Dorset, UK); Actin mouse monoclonal (Abcam, ab8226); normal rabbit IgG (12–370, Millipore, Billerica, MA).

### Immunofluorescence (IF)

RDEB and human control skin biopsies were frozen in optimal cutting temperature (OCT, Sakura Finetek USA, Torrance, CA) and cut at 6 microns on a cryostat. Sections were fixed with 50:50 methanol: acetone mixture for 5 minutes. Slides were rinsed with 1x PBS, permeabilized with PBS/0.1% Tween 20 (Sigma Aldrich, St. Louis, MO) for 5 minutes followed by blocking for 1 hour with PBST/3% BSA (Sigma Aldrich, St. Louis, MO). Primary antibodies were incubated for 1.5 hours at room temperature. Secondary antibodies donkey anti-rabbit cy3 (1:500, Jackson Immunoresearch, West Grove, PA), and Alexa Fluor 488 goat anti-mouse (1:1000, Molecular Probes Eugene, OR) were applied for 1 hour at room temperature. Slides were cover-slipped with hard set 4,6-diamidino-2-phenylindole (DAPI; Vector Labs, Burlingame, CA) and examined by confocal fluorescence microscopy (Olympus BX61, Olympus Optical, Tokyo, Japan).

### IF Quantification

Single color images were converted to grayscale and a grid was applied using Adobe® Photoshop C6 (Adobe Systems Incorporated, San Jose, CA). A uniform area was centered on the basement membrane at every intersection with the grid and pixel density was measured.

### Generation of a humanized mouse model of recessive dystrophic epidermolysis bullosa based on the common R578X nonsense mutation

In order to carry out preclinical testing to readthrough agents in the context of RDEB (Gartner U, Warbrick E and McLean WHI, unpublished) we generated a humanized mouse model where the human *COL7A1* cDNA carrying the commonly reported R578X nonsense mutation [44] was knocked into the murine Col7a1 locus by gene targeting in embryonic stem (ES) cells. Mouse generation was done under licence by TaconicArtemis GmbH, Cologne, Germany, using the gene targeting strategy as detailed in S1 Fig. The targeted allele included a small number of introns to improve expression *in vivo* and to help model nonsense mediated mRNA decay, a known feature of the human disease. Other than the R578X nonsense mutation (S1 Fig), no other changes were made to the human collagen VII coding sequence. Double selection of correctly targeted ES cell clones was performed, making use of a neomycin resistance cassette placed in intron 64 of the human gene as well as a puromycin resistance cassette placed immediately downstream of the human cDNA construct (Panels c and d in S1 Fig). Correct gene targeting was confirmed by a number of PCRs specific for the junctions of the targeted allele (not shown). Following generation of mice carrying the targeted allele shown in panel c of S1 Fig, both selection markers were removed by crossing to mice universally expressing Flp recombinase under control of the chicken-beta-actin promoter, to generate mice with the final humanized allele shown in panel d in S1 Fig.

Crossing of heterozygous humanized *COL7A1* mice gave the expected Mendelian ratios of wild-type, heterozygous and homozygous offspring. Wild-type and heterozygous littermates had no discernable phenotype, however, homozygous animals developed skin blistering, which was especially obvious on the forepaws, although blisters were also observed on other sites (S2 Fig). Histological examination of the skin of homozygous mice revealed blistering in the upper papillary dermis leading to complete sloughing off of the overlying epidermis (S3 Fig), completely mirroring the skin fragility observed in RDEB patients. Thus, these mice develop skin blistering that resembles both the human disease and the well-characterised *COL7A1* knockout mouse [45]. These animals are a suitable model for preclinical testing of new classes of drugs that act through suppression of nonsense mutations.

### Cells

S2 Table details all cells used in this study. Primary keratinocytes were isolated from fresh RDEB or healthy skin and cultured as previously described [30]. HpV immortalized cell lines have been described previously [46,47].

### Immunoblotting

Whole cell extracts were collected using radioimmunoprecipitation assay (RIPA) buffer and protein concentration determined by the Bradford Assay (Sigma Aldrich). Proteins were separated by electrophoresis using either NuPAGE 4–12% Bis-tris gels (Life Technologies, Paisley, UK) or 4–20% mini-PROTEAN TGX gels (Bio-Rad, Hertfordshire, UK) for type VII collagen. Protein was transferred to a nitrocellulose membrane using the Trans-Blot Turbo system (Bio-Rad). For conditioned media experiments 5x10^6^ cells were seeded per 100mm cell culture dish and allowed to adhere overnight in keratinocyte media containing serum. Cells were washed twice with PBS and 15ml keratinocyte media without serum added for 48 hours. Media was then transferred to a pre-chilled 50ml Falcon tube, spun at 1200rpm for 5 minutes to remove debris then concentrated to 200μl at 3200rpm at 4°C in an Amicon Ultra-15 Centrifugal filter unit with 10K cutoff (Millipore, Billerica, MA). Cells remaining on the plate were harvested in RIPA buffer as above and the volume of conditioned media loaded onto the gel normalized to the total protein amount from whole cell extracts.

### Co-immunoprecipitation

Protein extraction and co-immunoprecipitation were performed using the Universal Magnetic Co-IP kit (Active Motif, Rixensart, Belgium) according to manufacturer’s instructions. Briefly, cells were grown to confluence, proteins were extracted using whole cell lysis buffer and 200μg lysate incubated with either 1μl rabbit polyclonal sera to type VII collagen or a normal rabbit IgG control antibody (Millipore) for 2 hours at 4°C on a rotator. Protein G magnetic beads were then added for 1 hour at 4°C on a rotator and immune-complexes collected in 20μl 2x Laemmli buffer (+100mM DTT) after 5 washes in Co-IP wash buffer. 10μl sample was loaded per lane of a 4–20% mini-PROTEAN TGX gel (Bio-Rad) and western blotting performed as described above with antibodies to LH3 (mouse monoclonal, ProteinTech Group) and type VII collagen.

### Immunogold electron microscopy (IEM)

Normal human skin samples were processed for post-embedding immunoelectron microscopy as previously described [11,48]. Briefly, cryofixed, cryosubstituted samples were embedded in Lowicryl K11M resin and polymerized at -60°C under UV light. Selected blocks were used to produce ultrathin sections that were incubated with antibodies (80 μg/ml) diluted in PBS-based buffer and washed four times (5 minutes each). Further incubations were performed using rabbit anti-mouse and goat anti rabbit antibodies at 1 in 500 in PBS based buffer [11] followed by four washes and further incubation with either 5 or 15 nm gold-conjugated antibody for immunogold labeling (Biocell, Cardiff, UK) diluted 1 in 200 in Tris Buffered Saline. LH7:2 antibody which recognizes the N-terminal domain of human collagen VII [43] was included for comparison. Sections were viewed under a Hitachi H-7100 or Jeol 1010 transmission electron microscope (Hitachi, Jeol Tokyo, Japan) at 80 KV.

### Immunogold distribution assessment

The techniques for ultrastructural measurements were almost identical to those performed previously [11] using normal control human skin as the labeling substrate. Electron micrographs were taken at a standard magnification and enlarged by a standard factor. The final magnification was checked using electron micrographs taken of a carbon diffraction grating. For the purposes of standardization all measurements were made by one observer (JRM). All measurements were in nanometers (nm) from the plasma membrane (values were positive for measurements below the plasma membrane and negative for above the plasma membrane). The number of gold particles measured was between 200 and 300 (minimum 207 particles) for each antibody or antiserum (Table 1). Approximately 20 individual micrographs covering more than 40 μm of basement membrane were scanned and gold particle analysis performed for each epitope. Each antibody was labeled in duplicate using skin samples from two different individuals, and the data checked for any difference and then pooled. Control supernatant, pre-immune sera and an irrelevant isotype controlled (HLA) antibody were included as negative controls. Only non-obliquely (perpendicular) sectioned areas of basement membrane zone were included in the analysis with clearly defined lamina lucida and lamina densa. The dermal-epidermal junction beneath melanocytes or damaged areas was excluded. The distance from the plasma membrane to the center of each gold particle was measured and recorded. Very little gold labeling was localized further than 350nm from the plasma membrane zone. Gold particles that appeared to be to be clumped or associated with any stain-deposit were excluded from the analysis.

### Proximity Ligation Assay

Frozen skin sections were prepared as described above and protein interactions visualized by immunofluorescence using Duolink *in situ* PLA technology (Olink Bioscience, Uppsala, Sweden). Sections were blocked for 2 hours in 3% BSA/PBS then incubated with antibodies to LH3 (rabbit polyclonal, 1:10 dilution) and type VII collagen (mouse LH7.2, 1:800 dilution) overnight at 4°C. After washing, the slides were incubated with Duolink PLA Rabbit MINUS and PLA Mouse PLUS proximity probes (Olink Bioscience) at a 1:5 dilution at 37°C for 2 hours. Proximity ligation was then performed using the Duolink detection reagent kit (Olink Bioscience) according to manufacturer’s instructions except for the amplification step which was performed for 2 hours at 37°C. Slides were mounted using ProLong Gold Antifade with DAPI (Life Technologies, Paisley, UK) and imaged on the Zeiss Axioskop 2 fluorescent microscope using AxioVision Rel. 4.7 software (Carl Zeiss, GmbH, Jena, Germany).

### Retroviral transduction and siRNA transfection

*COL7A1* overexpression and siRNA knockdown were performed as described previously [46].

### Quantitative real time-PCR

cDNA was prepared from RNA isolated using RNA-Bee (AMS Biotechnology, Abingdon-on–Thames, UK) and the QuantiTect reverse transcription kit (Qiagen, Manchester, UK). For quantitative measurement of mRNA the QuantiTect SYBR Green PCR kit (Qiagen) was used with following primers: *PLOD3* forward (5’-CAGCTCCAGGACCACTTCTC-3’) and reverse (5’-ATGAGGATACGCAGGGTCTG-3’) and *GAPDH* forward (5’-TCCGGGAAACTGTGGCGTGA-3’) and reverse (5’-ACGGAAGGCCATGCCAGTGA-3’). PCR samples were set up on the QIAgility automated PCR workstation and reactions performed on the Rotor-Gene Q (Qiagen). Relative expression was calculated using the ΔΔ*C*_T_ method.

### Cell derived matrix and type VII collagen conditioned media incubation

The cell derived matrix experiment was performed essentially as described previously [46]. For incubation with type VII collagen conditioned medium, keratinocytes retrovirally transduced with *COL7A1* or the empty pBabe vector were seeded at 5x10^6^ cells per 100mm dish in keratinocyte medium plus serum. The next day the cells were washed twice with PBS and 15ml serum free keratinocyte medium containing 0.1 mmol/L l-ascorbic acid 2-phosphate (A8960; Sigma-Aldrich) was added for 48 hours. Media was concentrated, as detailed above, down to 1ml. Conditioned media was added to *COL7A1* null keratinocytes seeded in a 6 well plate and made up to a final volume of 3ml/well with fresh media. Cells were incubated for 48 hours at 37°C before protein extraction and analysis by western blot.

## Supporting Information

S1 Fig Gene targeting strategy to generate humanized COL7A1 R578X mutant mouse. (DOCX)

S2 Fig Homozygous transgenic animals exhibit skin blistering. (DOCX)

S3 Fig The skin of homozygous mice blisters at the dermal-epidermal junction. (DOCX)

S4 Fig PLOD3 mRNA is reduced in RDEB keratinocytes but not RDEB fibroblasts. (DOCX)

S5 Fig No change in type VII collagen expression after PLOD3 knockdown. (DOCX)

S1 Table Differentially expressed genes in cultured RDEB primary keratinocytes versus non-RDEB primary keratinocytes. (DOCX)

S2 Table Cells used in this study. (DOCX)
