# Supplementary material for: Lysyl Hydroxylase 3 Localizes to Epidermal Basement Membrane and Is Reduced in Patients with Recessive Dystrophic Epidermolysis Bullosa
Source: PLoS One. 2015 Sep 18;10(9):e0137639. doi: 10.1371/journal.pone.0137639 (PMC4575209; doi:10.1371/journal.pone.0137639)
Supplement: S1 Fig — (DOCX) [file pone.0137639.s001.docx]

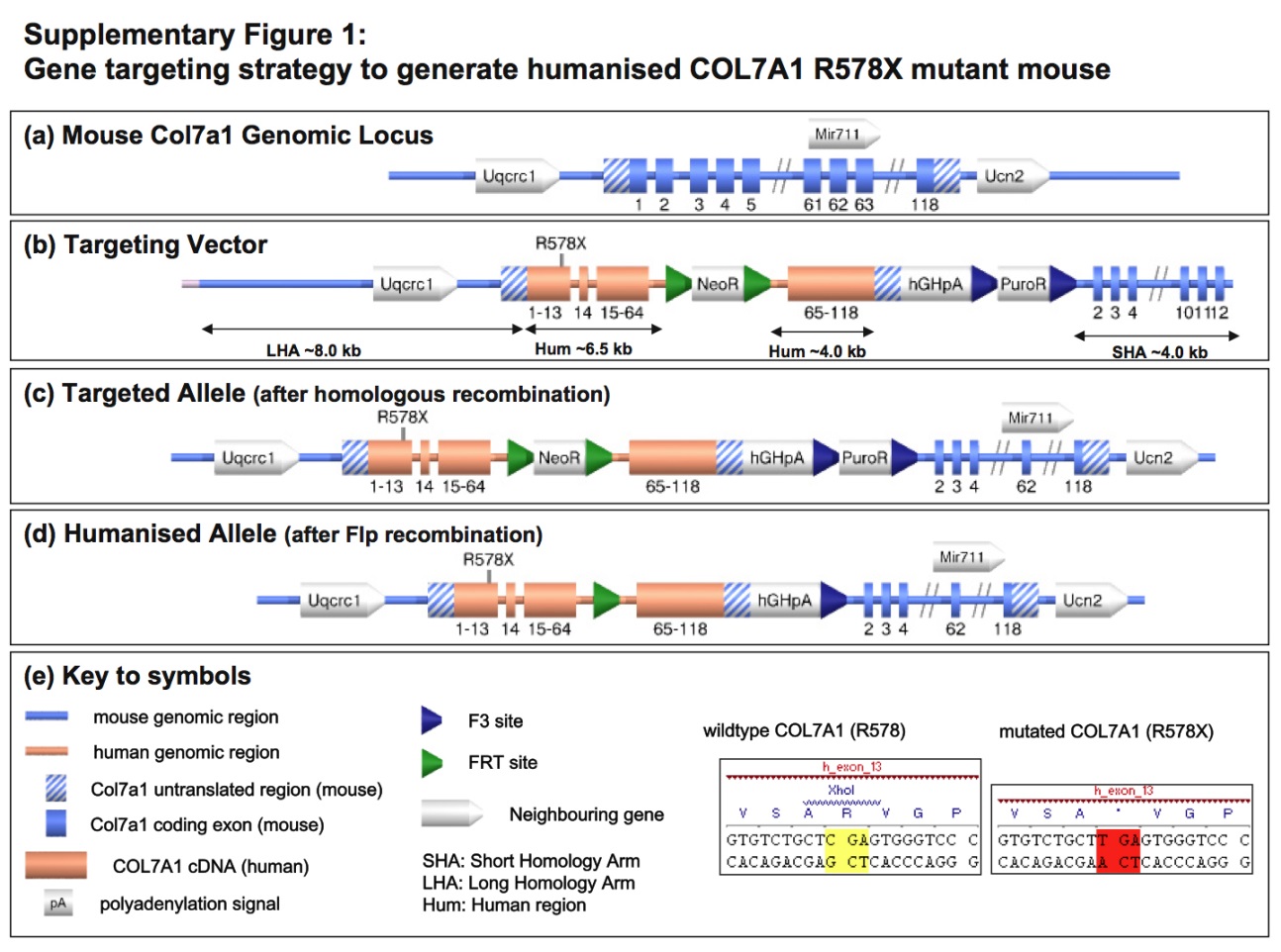


**S1 Fig. Gene targeting strategy to generate humanized COL7A1 R578X mutant mouse.**

(a) Schematic of the murine Col7a1 locus.

(b) Gene targeting vector for generation of the humanized allele. Exon 1 of the murine gene is replaced with the human COL7A1 cDNA carrying the R578X nonsense mutation and containing 3 introns (13, 14 and 64) to improve expression *in vivo* and allow for nonsense mediated mRNA decay. A neomycin resistance cassette, flanked by FRT recombination sequences (recognised by Flp recombinase), was placed within intron 64. A puromycin resistance cassette, flanked by F3 recombination sequences (also recognised by Flp recombinase) was placed downstream of the transcription unit. The native murine 5’ and 3’ UTR sequences were used, with addition of the human growth hormone polyadenylation signal downstream of the 3’UTR.

(c) Targeted allele following homologous recombination in mouse ES cells.

(d) Humanized allele following Flp recombination to remove neomycin and puromycin resistance cassettes.

(e) Key to symbols used in (a-d) plus the sequence of the R578X mutation.
