# Supplementary material for: Lysyl Hydroxylase 3 Localizes to Epidermal Basement Membrane and Is Reduced in Patients with Recessive Dystrophic Epidermolysis Bullosa
Source: PLoS One. 2015 Sep 18;10(9):e0137639. doi: 10.1371/journal.pone.0137639 (PMC4575209; doi:10.1371/journal.pone.0137639)
Supplement: S2 Fig — (DOCX) [file pone.0137639.s002.docx]

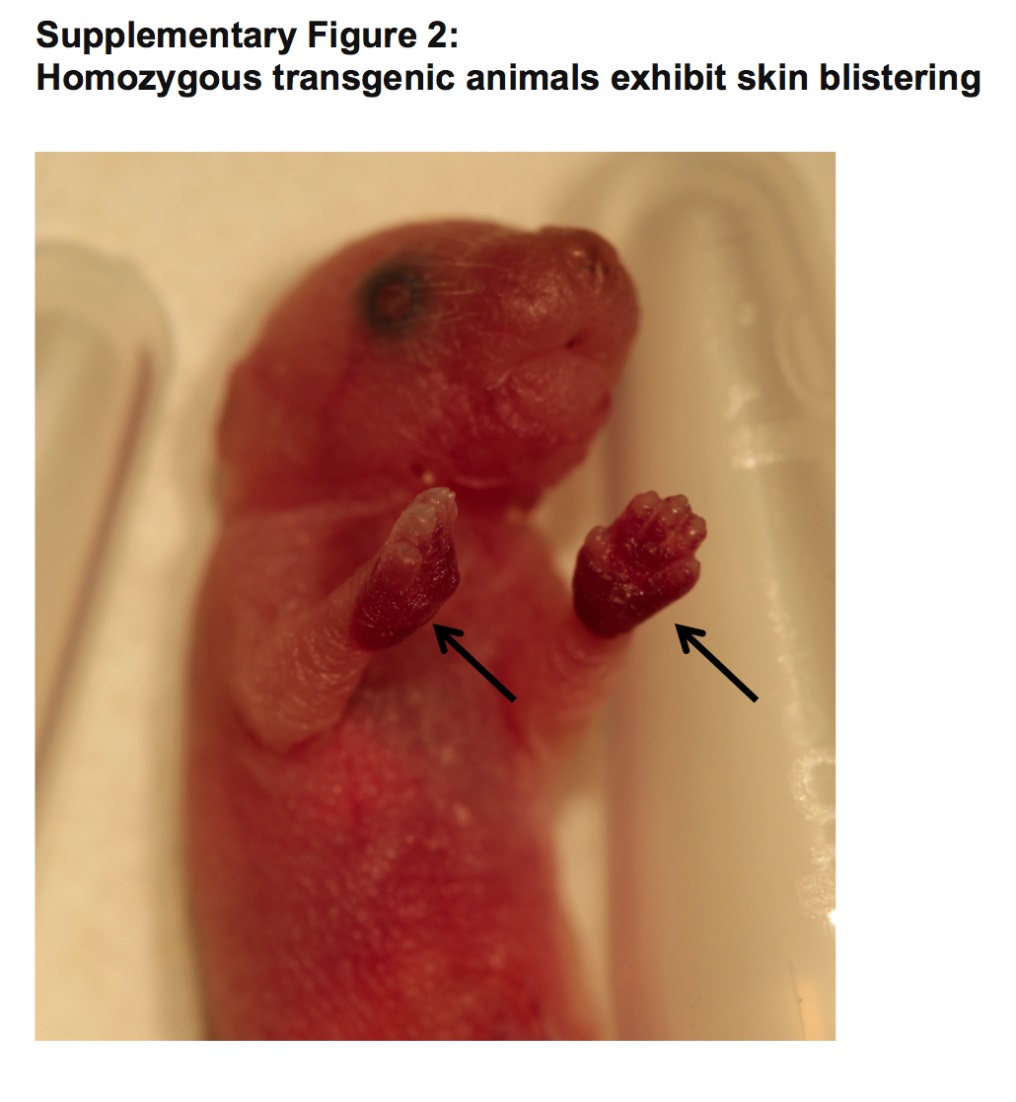


**S2 Fig. Homozygous transgenic animals exhibit skin blistering.**

Animals homozygous for the humanized COL7A1 R578X allele show overt skin blistering within the first few hours of birth, which was particularly readily observed on the forepaws. Arrows indicate blood-filled blisters on the forepaws.
