# Supplementary material for: Lysyl Hydroxylase 3 Localizes to Epidermal Basement Membrane and Is Reduced in Patients with Recessive Dystrophic Epidermolysis Bullosa
Source: PLoS One. 2015 Sep 18;10(9):e0137639. doi: 10.1371/journal.pone.0137639 (PMC4575209; doi:10.1371/journal.pone.0137639)
Supplement: S3 Fig — (DOCX) [file pone.0137639.s003.docx]

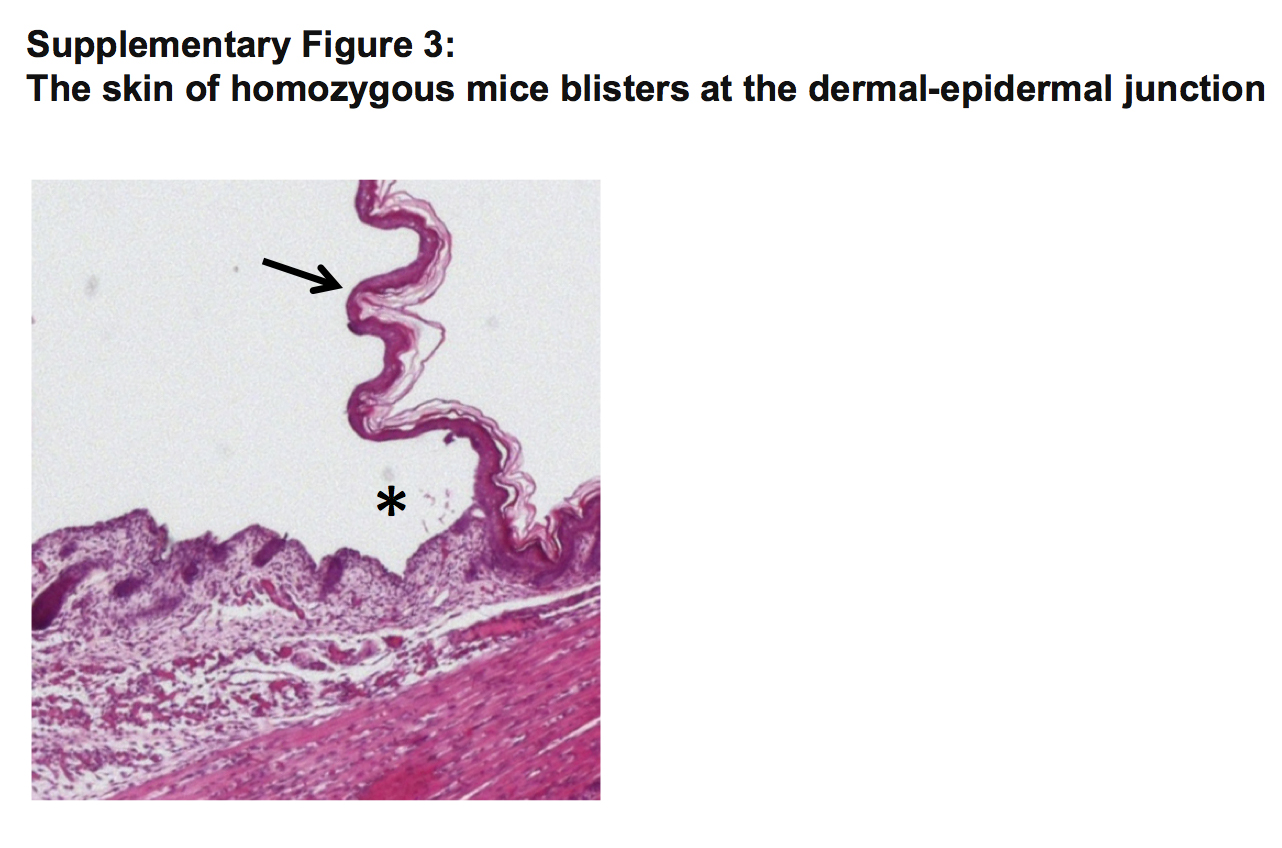


**S3 Fig. The skin of homozygous mice blisters at the dermal-epidermal junction.**

Histological analysis of the skin of mice homozygous for the humanized COL7A1 R578X allele shows tissue separation (*) at the dermal-epidermal junction, consistent with the human disease RDEB. Arrow indicates the separated epidermis of the blister roof.
