# Supplementary material for: Lysyl Hydroxylase 3 Localizes to Epidermal Basement Membrane and Is Reduced in Patients with Recessive Dystrophic Epidermolysis Bullosa
Source: PLoS One. 2015 Sep 18;10(9):e0137639. doi: 10.1371/journal.pone.0137639 (PMC4575209; doi:10.1371/journal.pone.0137639)
Supplement: S4 Fig — (DOCX) [file pone.0137639.s004.docx]

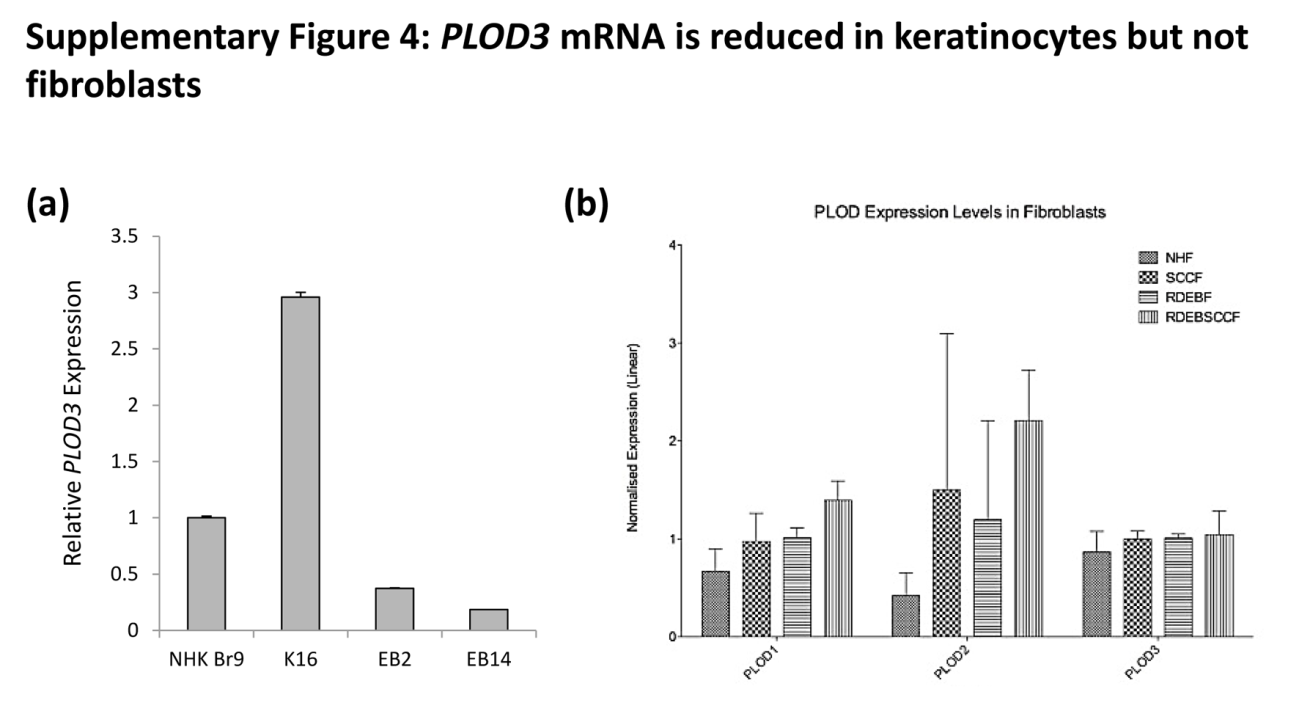


**S4 Fig. *PLOD3* mRNA is reduced in RDEB keratinocytes but not RDEB fibroblasts.**

1. Q-PCR relative to GAPDH shows reduction of *PLOD3* expression in RDEB keratinocytes (EB2, EB14) compared with normal keratinocytes (NHK Br9, K16)
2. Microarray data shows comparable *PLOD3* levels in primary fibroblasts isolated from normal skin (NHF), RDEB skin (RDEBF) and squamous cell carcinoma (SCCF and RDEBSCCF). Microarray data was previously generated from cultured fibroblasts as described [28]. The data are deposited in NCBI's Gene Expression Omnibus [49] accessible through GEO Series accession number GSE37738 (<http://www.ncbi.nlm.nih.gov/geo/query/acc.cgi?acc=GSE37738>).

Graph shows normalised average signal intensities for single probes representing the *PLOD1*, *PLOD2* and *PLOD3* genes for each sample group. *PLOD1* mRNA shows a slight increase in RDEBF compared with NHF which is not statistically significant. *PLOD2* mRNA increases in RDEBF and SCCF compared with NHF. This is in keeping with an increase in TGFbeta signalling observed in RDEBF [28,29] and SCCF [28] and TGFbeta regulation of *PLOD2* but not *PLOD1* or *PLOD3* [50].

**Additional References**

49. Edgar R, Domrachev M, Lash AE (2002). Gene Expression Omnibus: NCBI gene expression and hybridization array data repository. Nucleic Acids Res 30: 207-210.

50. Witsch TJ, Turowski P, Sakkas E, Niess G, Becker S, Herold S et al (2014). Deregulation of the lysyl hydroxylase matrix cross-linking system in experimental and clinical bronchopulmonary dysplasia. Am J Physiol Lung Cell Mol Physiol 306: L246-259.
