# Supplementary material for: Lysyl Hydroxylase 3 Localizes to Epidermal Basement Membrane and Is Reduced in Patients with Recessive Dystrophic Epidermolysis Bullosa
Source: PLoS One. 2015 Sep 18;10(9):e0137639. doi: 10.1371/journal.pone.0137639 (PMC4575209; doi:10.1371/journal.pone.0137639)
Supplement: S5 Fig — (DOCX) [file pone.0137639.s005.docx]

**S5 Fig. No change in type VII collagen expression after *PLOD3* knockdown.**

Levels of type VII collagen do not change after siRNA depletion of *PLOD3*, encoding LH3, (denoted by “Si”) as compared with non-targeting control (“NT”) as determined by Western blotting.
