# Supplementary material for: Lysyl Hydroxylase 3 Localizes to Epidermal Basement Membrane and Is Reduced in Patients with Recessive Dystrophic Epidermolysis Bullosa
Source: PLoS One. 2015 Sep 18;10(9):e0137639. doi: 10.1371/journal.pone.0137639 (PMC4575209; doi:10.1371/journal.pone.0137639)
Supplement: S1 Table — (DOCX) [file pone.0137639.s006.docx]

**S1 Table: Differentially expressed genes in cultured RDEB primary keratinocytes versus non-RDEB primary keratinocytes.** Analysis of previous microarray data comparing normal primary keratinocytes (n=5) with primary RDEB keratinocytes (n=4) identifies 82 differentially expressed genes based on average signal intensity and Student t-Test significance ([Watt et al 2011](#_ENREF_4)).

**
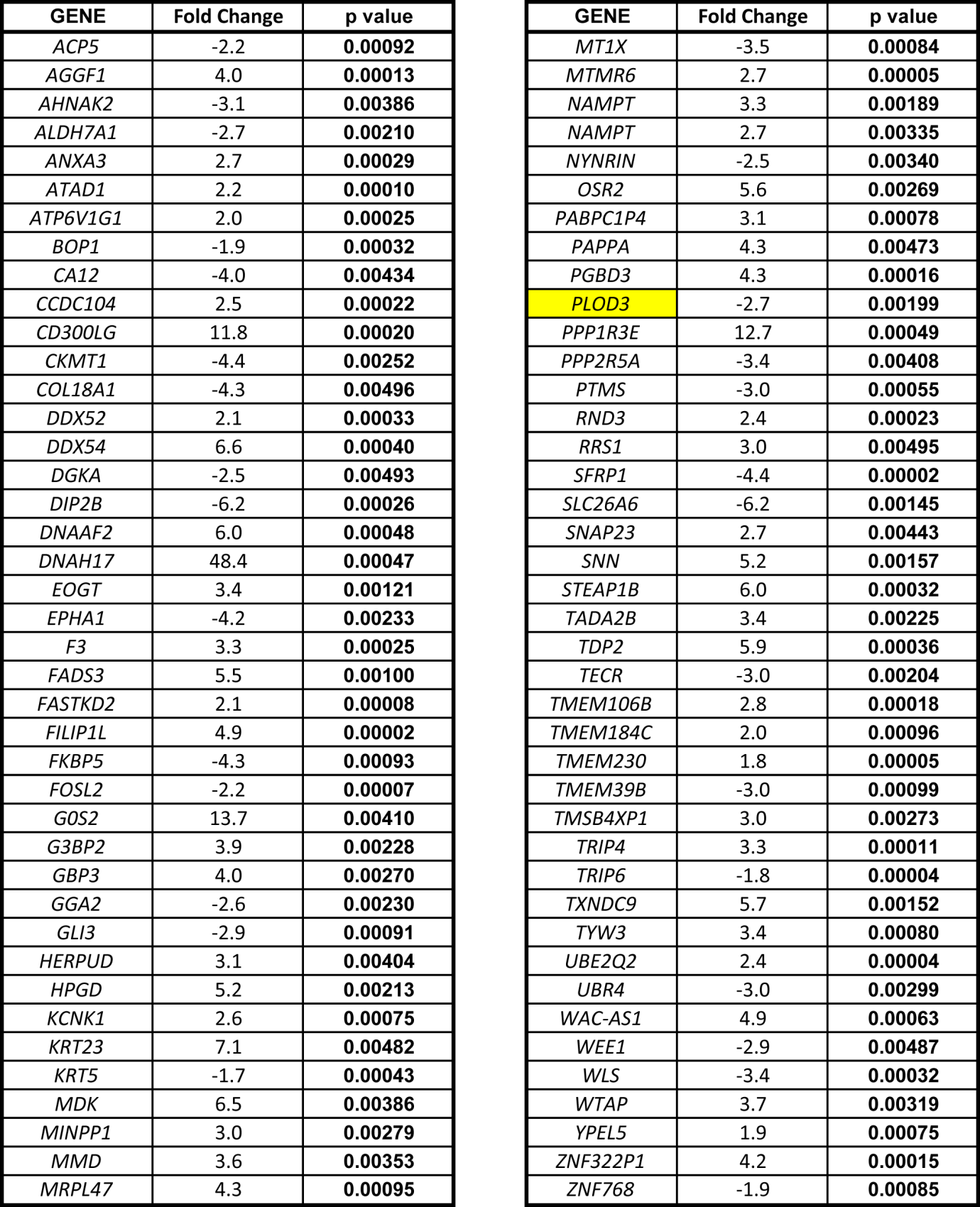
**
