# Supplementary material for: Lysyl Hydroxylase 3 Localizes to Epidermal Basement Membrane and Is Reduced in Patients with Recessive Dystrophic Epidermolysis Bullosa
Source: PLoS One. 2015 Sep 18;10(9):e0137639. doi: 10.1371/journal.pone.0137639 (PMC4575209; doi:10.1371/journal.pone.0137639)
Supplement: S2 Table — (DOCX) [file pone.0137639.s007.docx]

**S2 Table: Cells used in this study**
